# Body image before and after coronary artery bypass graft surgery: comparison and its contributing factors

**DOI:** 10.1186/s40359-020-00451-z

**Published:** 2020-08-03

**Authors:** Mohsen Adib-Hajbaghery, Sedigheh Miranzadeh, Mahsa Tahmouresi, Ismail Azizi-Fini

**Affiliations:** 1 Trauma Nursing Research Center, Kashan University of Medical Sciences, Kashan, Iran; 2 Medical Surgical Nursing Department, Kashan University of Medical Sciences, Kashan, Iran

**Keywords:** Body image, Coronary artery disease, Coronary artery bypass graft surgery, Nursing, Iran

## Abstract

**Background:**

Cardiovascular diseases are the leading cause of death in the world. Coronary artery bypass graft (CABG) surgery is among the treatment options for coronary artery disease. However, it is associated with significant physical and psychological problems. This study sought to compare body image before and after the surgery and to determine its contributing factors.

**Methods:**

This comparative study was conducted in 2017 on a sample of 140 patients consecutively recruited from Shahid Beheshti hospital, Kashan, Iran. Body image was assessed before and 4 weeks after the surgery (T1 and T2) using Multidimensional Body-Self Relations Questionnaire. The independent-sample and paired *t* tests, one-way analysis of variance, Pearson correlation test, and multiple regression were conducted for data analysis.

**Results:**

Participants’ mean score of body image was 139.60 ± 13.21 at T1 and 160.25 ± 7.75 at T2 and the variation was statistically significant (*p* = < 0.001). At T1, only the three factors of age (*p* = 0.005), education at high school diploma and higher levels (*p* < 0.001), and being housekeeper (*P* = 0.048) could significantly explain BI (R^2^ = 0.231). However, at T2, none of the factors were significant predictors for BI (*P* > 0.05).

**Conclusions:**

Candidates for CABG have poor body image. After the surgery, their body image improves significantly. Healthcare providers need to employ programs to improve body image among these patients.

## Background

Cardiovascular diseases are of the most common life-threatening health conditions [1] with a progressively increasing global prevalence. With more than 90,000 deaths, cardiovascular diseases accounted for 25% of all deaths in Iran in 2016 [2, 3].

Coronary artery bypass graft (CABG) surgery is one of the most principal treatments for serious coronary artery disease [4]. Around 35,000–50,000 CABG surgeries are annually performed in Iran [4, 5]. Although CABG is a safe and effective technique, it has considerable adverse psychological effects [6].

Studies on patients with CABG showed that they suffer from a variety of problems such as preoperative and postoperative anxiety and stress [7], delirium, sleep problems [8, 9], role limitations and mental problems [10], low quality of life and body image (BI) alterations [11–13].

Patients interpret cardiac surgery as an event associated with disablement and changes in BI. For many patients, it is a threatening procedure that might results in increased anxiety [14]. Disfigurement due to the scares resulted from the chest surgery may disturb how individuals perceive and value their bodies, causing them to experience body change as a psychologically threatening situation [15].

BI has been defined in several ways. A commonly used definition suggests that BI consists of perceptions, thoughts, and feelings associated with the body and bodily experiences [16]. BI is a multidimensional construct [17]. Its formation starts at birth; develops as the person grows up, and changes during the different stages of life [18].

The altered BI and its subsequent psychological effects might lead to social stigmatization, insecurities in identity, lowered self-esteem, heightened emotional tension and feelings of sexual unattractiveness [15, 17, 19]. Also, altered BI after some surgeries can lead to psychological trauma. Disfigurement of the body due to the chest scars may also negatively affect how people perceive themselves [13]. People with a more realistic BI are more likely to have better self-esteem and less anxiety and depression [20].

A number of studies have investigated the BI and related factors in different groups of people, especially girls and women. Some studies reported that a majority of adults and college-age women experience degrees of dissatisfaction with their bodies, and poor BI was associated with sociocultural factors and specially affected by peer and media [21, 22] A number of studies also investigated the factors affecting BI of college students and reported that body surveillance, body shame, sociocultural attitudes toward appearance, self-esteem [23], exposure to social media such as Facebook and Instagram, and the time per day spent on using social networking services significantly affected the students’ BI [24]. Another study also reported that most of the community-dwelling older adults were dissatisfied with their BI, and BI satisfaction was correlated with their functional abilities and muscular strength [25]. A study on millennial females also reported that personal preferences, morals and beliefs, and certain occasions, seasons, climate, and the weather can significantly affect their BI satisfaction [26]. A recent study also reviewed Iranian people’s BI and some of its contributing factors and reported that BI dissatisfaction is prevalent in different ages and social groups. BMI, social pressures, and self-steam were among the most influential factors affecting BI in the Iranian population [27].

There are several instruments for the assessment of BI in different groups of people. Using different instruments, a number of studies have investigated BI in different patients. For instance Bagheri and Mazaheri used the Multidimensional Body-Self Relations Questionnaire (MBSRQ) to investigate BI in patients with breast cancer [28] Goudarzian et al. also used Body Shape Questionnaire (BSQ-34) to assess BI in patients with congestive heart failure [29]. İyigün et al. also used the Body Image Questionnaire (BIQ) to compare BI before and after cardiac surgeries [13]. In another study, Mojallal et al. used the MBSRQ to assess BI in patients who undergone cosmetic surgeries [30]. A recent systematic review has critically appraised the properties of common BI assessment instruments and concluded that the majority of the instruments are reliable and valid, although suitability varied across populations, and some of the instruments still need more evaluations [31]. The MBSRQ is one of the most widely used questionnaires in Iran and showed suitable validity and reliability in different Iranian populations [20, 32]. While some of the BI instruments focus on a particular aspect of BI, the MBSRQ is multidimensional and assesses many aspects of BI [33]. It is able to differentiate between the evaluation of appearance-related aspects and the person’s orientation toward these aspects (i.e., the perceived importance of appearance and its influence on the person’s behavior) [34].

Few studies are available on BI after cardiac surgery. In a study, İyigün et al., investigated the BI in patients who undergone cardiac surgery and reported that robot-assisted surgery has advantages in terms of BI, self-esteem, and cosmetic outcomes over the conventional open heart surgery [13]. Another study also reported that scars resulting from cardiac surgery may considerably affect a patient’s BI and several aspects of daily life [15]. A study also reported that BI has improved over time after surgery [35]. A number of studies also investigated the factors affecting postsurgical BI and reported conflicting results. For example, in a study no significant relationship was found between BI and variables such as age, sex, marital status, educational status, income, and occupation [36]. However, another study reported that male patients had better BI than females after obesity surgery [37].

Patients with coronary artery occlusion who are candidates for cardiac surgery have certain activity limitations before surgery and are unable to perform much of their normal routines. These limitations affect their BI [13]. If patients have appropriate hemodynamic conditions, they are encouraged to go out of the bed in the first days after surgery. Usually, they are discharged from the hospital 1 week after surgery and undergo cardiac rehabilitation, and then return to their daily lives [12]. It is supposed that their return to daily life in the first weeks after surgery may improve their BI. So the question arises, “Is there a difference between the BI before surgery and one month later in patients undergoing cardiac surgery?” Given the contradictory results of the studies and the lack of related studies in Iran, two questions still need to be answered: 1) what is the difference between a BI score before and 1 month after CABG? 2) How do individual and clinical characteristics of patients affect their BI score? Therefore, this study sought to 1) compare BI scores before and 1 month after CABG, and 2) to determine the effect of some individual and clinical characteristics of patients on their BI score.

## Methods

### Design and participants

**Fig. 1:**
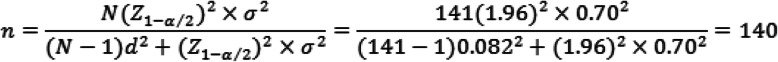
Sample size calculation

This comparative study was conducted on 140 patients with CABG. Sample size was determined based on the findings of a former study [38] and with a standard deviation of 0.7, a type I error of 0.05, and a measurement precision (*d*) of 0.082 (Fig. 1).

$$ n=\frac{N{\left({Z}_{1-\alpha /2}\right)}^2\times {\sigma}^2}{\left(N-1\right){d}^2+{\left({Z}_{1-\alpha /2}\right)}^2\times {\sigma}^2}=\frac{141{(1.96)}^2\times {0.70}^2}{\left(141-1\right){0.082}^2+{(1.96)}^2\times {0.70}^2}=140 $$

Eligible patients were consecutively recruited from the cardiac surgery unit of Shahid Beheshti hospital, Kashan, Iran. Eligibility criteria were CABG candidacy based on angiographic findings, no active psychiatric disorder as determined by psychiatrist, no psychoactive agent use, no obvious BI-affecting physical disorder (such as burn scars or limb amputation), and agreement for participation. Exclusion criteria were voluntary withdrawal or death during the study.

### Instruments and data collection

Two instruments were used for data collection. The first was a demographic and clinical characteristics questionnaire with items on age, gender, education level, marital status, employment status, tranquilizer use, body mass index (BMI), and other comorbidities. The second instrument was the 46-item MBSRQ that developed by Brown et al. in 1990 for BI assessment [34]. MBSRQ included six dimensions, namely appearance evaluation (AE, seven items), appearance orientation (AO, 12 items), fitness evaluation (FE, three items), fitness orientation (FO, 13 items), subjective weight (SW, two items), and body areas satisfaction (BAS, nine items). Items were scored from 1 (Completely disagree) to 5 (Completely agree). Some items are negatively worded and hence are reversely scored. The possible total score of MBSRQ was 46–230, with greater scores showing better BI. A former study reported that the Cronbach’s alpha values of the questionnaire and its dimensions were 0.84 and 0.66–80, respectively [39]. In the current study the overall Cronbach’s alpha was calculated as 0.81 and 0.68–0.79 for different subscales.

Data were collected 1 day before CABG (T1), and 1 month after it (T2). As some patients were illiterate, data collection was done through individual structured interviews in a private environment. The first measurement was done during the hospital stay of the participants, but the follow-up data collection was done at the outpatient cardiac rehabilitation center of the hospital; when the patients referred for their follow-up and rehabilitation.

### Ethical considerations

This study has the ethical approval of the Ethics Committee of Kashan University of Medical Sciences, Kashan, Iran (approval code: IR.KAUMSREC.1396.23). We informed patients about the study aim, guaranteed the confidentiality of their data, and asked them to sign an informed consent form. All the questionnaires were anonymous and all the patients were informed that their participation was voluntary.

### Data analysis

Data were analyzed via the SPSS for Windows software (v. 16.0). The measures of descriptive statistics were used to describe participants’ characteristics and MBSRQ scores. The independent-samples *t* test and the one-way analysis of variance (ANOVA) were conducted to compare the mean MBSRQ scores between the participants’ subgroups. The paired *t* test was used to compare the mean MBSRQ scores and its dimensions before and 1 month after the surgery. Pearson correlation coefficient was used to examine the correlation between BI score, age and BMI. Moreover, multiple regression analysis was used to examine the effects of demographic and clinical characteristic on BI. To this end, first, the backward model was conducted with the removal criterion of *P* > 0.20. Then, all the remaining variables with *P* < 0.20 were again entered into the model and analyzed using the forward method. Before we perform the multiple regression analysis, the categorical variables were first converted to dummy variables to represent subgroups of the samples. Also, to enter the ordinal variables to the model, we coded them as 0, 1, 2, and so on. Age and BMI entered the model as continuous variables. *P* values< 0.05 were considered significant.

## Results

All patients were followed until the end of the study and no one was excluded. Among 140 participants, 53.6% were male, 87.9% were married, 57.1% had secondary education, 34.3% were retired, 73.6% had a history of other comorbidities (such as hypertension: 14.4% and diabetes mellitus: 15.7%), and 95.7% had a BMI over the normal range (Table 1). The mean age of the participants was 55.71 ± 4.52 years. The mean score of BI was 139.60 ± 13.21 at T1 and 160.25 ± 7.75 at T2 (*P* < 0.001). Moreover, at T2, the participants’ mean scores have significantly been increased (*P* < 0.001) in all dimensions of BI (especially in the fitness orientation dimension) except for the subjective weight that has been decreased (*P* < 0.001) (Table 2).

**Table 1:** Participants’ body image scores at different measurement time points based on their characteristics

Demographics characteristics	N (%)	Before surgery	***P*** value	After 1 month	***P*** value
		Mean ± SD		Mean ± SD	
**Gender**			0.579^a^		0.110^a^
Male	75 (53.6)	139.2 ± 11.94		161.22 ± 6.44	
Female	65 (46.4)	141. 6 ± 140.27		159.12 ± 8.92	
**Marital status**			0.002^a^		0.533^a^
Married	123(87.9)	138.30 ± 12.31		160.09 ± 7.64	
Single	17(12.1)	149.00 ± 15.96		161.35 ± 8.65	
**Age (Years)**			< 0.001^b^		0.886^b^
40–50	33(23.6)	146.12 ± 16.05		159.66 ± 8.85	
51–60	89(63.3)	138.58 ± 12.11		160.43 ± 7.65	
> 60	18(12.9)	132.72 ± 6.71		160.38 ± 6.29	
**Educational status**			< 0.001^a^		0.587^a^
Illiterate	11 (7.9)	137.18 ± 15.40		158.54 ± 6.97	
Elementary education	14 (10)	136.57 ± 5.40		162.21 ± 5.89	
Secondary education	80 (57.1)	135.16 ± 9.98		159.97 ± 6.92	
High school diploma and higher ^c^	35(25)	149.22 ± 15.68		161.08 ± 9.90	
**Employment**			< 0.001^b^		0.620^b^
White-collar worker	13(9.3)	153.23 ± 14.78		161.69 ± 7.00	
Self-employed	34(24.3)	138.70 ± 11.03		161.32 ± 6.35	
Housewife	45(32.1)	140.50 ± 14.12		159.33 ± 9.38	
Retired	48(34.3)	135.40 ± 10.65		159.95 ± 7.21	
**Tranquilizer use**			0.032^a^		0.332^a^
Yes	36(25.7)	135.55 ± 8.53		161.33 ± 7.09	
No	104(74.3)	141.00 ± 14.26		159.87 ± 7.96	
**History of co-morbidities**			0.185^a^		0.567^a^
Yes	103(73.6)	138.71 ± 13.08		160.47 ± 7.37	
No	37(26.4)	142.08 ± 13.46		159.62 ± 8.81	
**Type of co-morbidities**			0.170		0.563
Hypertension	20(14.4)	142.58 ± 13.34		160.02 ± 7.82	
Diabetes mellitus	22(15.7)	135.08 ± 9.94		160.30 ± 5.78	
Hyperlipidemia	17(12.1)	134.84 ± 8.78		159.78 ± 6.58	
Other diseases	44(31.4)	139.51 ± 15.24		158.23 ± 6.15	
**Body mass index**			0.073^b^		0.163^b^
18.5–24.9	6(4.3)	143.16 ± 9.66		161.83 ± 7.60	
25–29.9	72(51.4)	141.18 ± 14.53		161.12 ± 8.51	
30–34.9	52(37.1)	139.86 ± 11.33		159.76 ± 6.82	
35–39.5	10(7.1)	130.80 ± 10.89		155.50 ± 5.01	

^a^The results of the independent-sample *t* test

^b^The results of the one-way ANOVA

^c^Tukey post hoc analysis showed that only patients with ‘diploma and higher education’ differed significantly from other groups

**Table 2:** Comparisons of the variations of the mean scores of BI and its dimensions across the three measurement time points^a^

Body image dimensions	Time point		95% Confidence Interval of the Difference	***P*** value ^**b**^
	Before	After 1 month		
**Total**	139.60 ± 13.21	160.25 ± 7.75	18.05, 23.22	< 0.001
**Appearance evaluation**	21.85 ± 2.97	24.27 ± 2.41	1.76, 3.06	< 0.001
**Appearance orientation**	35.47 ± 4.72	41.29 ± 3.18	4.94, 6.68	< 0.001
**Fitness evaluation**	7.86 ± 1.69	10.12 ± 1.49	1.84, 2.66	< 0.001
**Fitness orientation**	36.52 ± 4.99	45.59 ± 3.41	8.12, 10.01	< 0.001
**Subjective weight**	7.26 ± 1.58	6.30 ± 1.25	−1.30, −0.61	< 0.001
**Body areas satisfaction**	30.62 ± 4.39	32.66 ± 2.86	1.16, 2.92	< 0.001

^a^Data presented as Mean ± SD

^b^The results of the paired t test

As Table 1 shows, at T1, single participants (*P* = 0.002), patients who aged 40–50 (*P* < 0.001), those with high school diploma and university degrees (*P* < 0.001), those who were white-collar employees (*P* < 0.001), and patients who did not use tranquilizers (*P* = 0.032) had greater BI scores than patients in other subgroups. However, none of the participants’ characteristics significantly affected their BI mean score at the second measurement 1 month after the surgery (*P* > 0.05).

Pearson correlation test showed a significant correlation between BI scores and age at T1 (*r* = − 0.35, *P* < 0.001) but the correlation was not significant at T2 (*r* = 0.13, *P* = 0.16). However, the correlation between BI scores and BMI was not statistically significant neither at T1 (*r* = − 0.095, *P* = 0.37) nor at T2 (*r* = − 0.12, *P* = 0.21). Multiple regression analysis showed that at T1, only the three factors of age (*P* = 0.005), education at high school diploma and higher levels (*P* < 0.001), and being housekeeper (*P* = 0.048) could significantly explain BI. These three variables could explain 23% of the variance of the BI score (adjusted R^2^ = 0.231, Table 3). However, at T2, none of the factors could significantly predict BI (*P* > 0.05).

**Table 3:** The results of the regression analysis for the predictors of body image at T1

Model	Unstandardized Coefficients		Standardized Coefficients		***P*** value
	B	Std. Error	Beta	t	
**(Constant)**	175.422	13.188		13.302	< 0.001
**High school diploma and higher**^**a**^	9.721	2.436	0.320	3.992	< 0.001
**Age**	−0.662	0.232	−0.227	−2.850	0.005
**Housekeeper**^**b**^	−4.243	2.124	−0.150	−1.998	0.048

R = 0.248, Adjusted R^2^ = 0.231

^a^Secondary education

^b^Reference category: Retired

## Discussion

The present study showed that our participants possessed 60.7% of the possible BI score at the beginning but their mean overall BI score increased about 9% at the end of the study. Although we did not assess the patients’ mood or physical problems, however, the relatively low BI score might be attributable to the physical problems these patients experience due their cardiac problem that might negatively affect their mood and BI. In a former study, the patients possessed about 77% of the possible BI score before open heart surgery but their mean BI score decreased about 7% after surgery and the researchers attributed the decreased score to the psychological trauma experienced due to the chest scars [13]. The low BI before surgery can be attributed to patients’ poor preoperative mood and psychological status as well as fear and anxiety over surgery and death [14]. A recent review study also confirmed the associations between BI dissatisfaction and anxiety and depression [40]. Some studies have also reported that most of the patients hospitalized for CABG experience various emotions ranging from a slight fear to anxiety, depression, and fear of death, and these problems are more annoying than their chest pain [41], which consequently can affect BI [42]. A study of BI in patients receiving hemodialysis and those undergone renal transplantation has also reported that changes in the body appearance and functions, can affect BI, self-concept, and personal identity [43]. However, improvements in physical conditions, reduction of cardiac problems [44], greater ability to perform the activities of daily living [45], and better psychological status [46] might play roles in the better BI 1 month after surgery.

Findings also indicated that male and female participants did not significantly differ from each other respecting BI mean score both at T1 and T2. A number of studies reported that gender has a considerable impact on people’s body image [47]. Females are usually more sensitive to their body image than males [48]. However, a study on patients who underwent bariatric surgery has reported that males and females did not differ in their body image dissatisfaction both before and after surgery [49]. Nonetheless, further studies are yet necessary into the association of gender and BI after major surgeries such as CABG.

Another study finding was better BI among participants with higher education levels than those with lower education at T1 and no significant difference between them at T2. Previous studies on patients after CABG surgery [12] and on the general population [50] also reported that those with higher education levels have better BI. This finding may be due to the greater level of health-related knowledge and greater coping abilities among participants with higher educational status [51]. However, the insignificant difference between BI scores of people with high and low education level at T2 can be attributed to the improvements in knowledge, perceptions, and attitudes about CABG and self-care among all patients as a result of experiencing the surgery and receiving self-care educations during the postoperative period.

We also found poorer BI among older participants at T1. However, at T2, older and younger participants did not significantly differ respecting BI. An earlier study also reported that older patients have lower BI score [52]. Perhaps, before the surgery, older adults are more anxious about their future and the outcome of surgery [53]. However, their anxiety might be alleviated after CABG.

Another study finding was better BI among single participants compared to their married counterparts at T1 and no significant difference between them at T2. There are conflicting evidence about the association between marital status and BI. A study reported that married men and women reported a more positive BI than single ones [54]**.** In contrast, a study showed that single girls had better BI in comparison with married women [50]. However, another study could not find a relationship between marital status and BI [55]. Although a study reported that marriage decreases the importance of body appearance [56]. However, poorer BI among married participants in our study might be attributable to their worries about their future, and concern about their marital relationships, spouses, and children. Nonetheless, due to the minority of single patients in the present study, further studies are needed to provide firmer evidence respecting the effect of marital status on CABG candidates’ BI.

The study also showed that at T1, participants who were white-collar employees had better BI than other participants. However, at T2, no significant difference was found in this regard.

A previous study found no significant association between BI and employment [57], but another study confirmed the positive effect of employment on body satisfaction [58]. Perhaps, employed people and especially white-collar employees have better job security, and lower concerns over income and employment, and hence, have more positive attitudes towards life and better perception about self [53, 59, 60]. However, no significant difference among occupational groups respecting BI at T2 may probably be due to the decreases in the levels of stress and anxiety and subsequent improvements in BI.

Regression analysis also showed that age, education at high school diploma and higher levels, and being housekeeper were associated with BI at T1. The significant effects of these factors at T1 can be attributed to the effects of these factors on patients’ health literacy, attitudes, anxiety, and psychological status. However, at T2, none of the study variables significantly predicted BI and this finding is attributable to the improvements in physical and psychological condition of patients at this time.

## Limitations

One limitation of the present study was that the study was conducted in a single hospital setting. Large-scale multicenter studies are recommended to compare the variations of BI during pre- and post-CABG periods. Moreover, regression analysis showed that study variables explained the variance of BI by only 23% at T1. This means that around 70% of the variance has been related to factors not assessed in this study. Future studies are recommended to assess the effects of other factors such as patients’ mood, stress, anxiety, and social and familial support on BI.

## Conclusion

This study suggests that BI is poor before CABG and significantly improves after 1 month Moreover, CABG candidates with younger ages, higher educational degrees, and white-collar employment had better BI before the surgery. Therefore, nurses and other healthcare providers need to employ programs for improving CABG candidates’ knowledge about CABG, its preoperative and postoperative care, its complications, and relevant self-care activities.

## Data Availability

The datasets used and/or analyzed during the current study are available from the corresponding author on reasonable request.

## Abbreviations

BI: Body image
CABG: Coronary artery bypass graft
T1: Time 1
T2: Time 2
